# Exploring the clinical efficacy of different nonsurgical rehabilitation interventions for humeral lateral epicondylitis: A protocol for network meta-analysis

**DOI:** 10.1097/MD.0000000000030234

**Published:** 2022-08-26

**Authors:** Qing Wan, Qin Lan, Fang Zhi, Xingzhen Lin

**Affiliations:** a Nanchang Hongdu Hospital of Traditional Chinese Medicine, Nanchang, Jiangxi Province, China.

**Keywords:** humeral lateral epicondylitis, network meta-analysis, nonsurgical rehabilitation interventions

## Abstract

Lateral epicondylitis is a common musculoskeletal disorder characterized by the decreased grip and upper limb strength and pain in the lateral aspect of the elbow joint. At present, shock wave, corticosteroid injection, platelet-rich plasma injection, and Dextrose prolotherapy therapy nonsurgical rehabilitation treatment methods are commonly used in clinical. However, the conclusions of studies comparing treatment efficacy between them are controversial, with most studies focusing on limited comparisons and a lack of direct and indirect comparisons between them, making it difficult to have a clearer and more comprehensive understanding of the efficacy between them.

**Purpose:**

To comprehensively compare the effectiveness of shockwave, corticosteroid injection, platelet-rich plasma injection, and Dextrose prolotherapy therapy for the treatment of external humeral lateral epicondylitis using a reticulo-meta-analysis.

**Methods:**

Randomized controlled trials on the effectiveness of shock wave, corticosteroid injection, platelet-rich plasma injection, and Dextrose prolotherapy therapy for the treatment of external humeral lateral epicondylitis were searched in PubMed, The Cochrane Library, CNKI, and Wan-Fang databases for the period January 1, 2016 to January 1, 2021 for each database. Two investigators independently screened the literature, extracted data according to inclusion and exclusion criteria, and evaluated the quality of the literature in parallel. Statistical analyses were performed using Stata 14.0 software to compare differences in efficacy between treatment measures using ratio and 95% confidence interval as effect indicators and to rank efficacy.

**Results:**

Nine randomized controlled trials with a total of 289 patients with external humeral lateral epicondylitis were included, involving 4 nonsurgical rehabilitation measures and 6 intervention options. Quadrilateral ring to test the inconsistency of each closed-loop study finding, and the results show that the inconsistency factor was bounded at 1.65, with lower 95% confidence interval of 0.47 and 2.84 for both, which in summary indicates that the formation between the treatment measures in this study indicates that each closed-loop inconsistency was good. The SUCRA curve showed that platelet-rich plasma injection + shockwave was the first treatment with an area under the curve of 86.9%. Six treatment measures were ranked as follows: platelet-rich plasma injection + shockwave > platelet-rich plasma injection > shockwave > corticosteroid > corticosteroid + shockwave > prolotherapy.

**Conclusions:**

It is believed that in the course of clinical practice, platelet-rich plasma injection combined with shockwave therapy can be preferred for patients with humeral epicondylitis.

## 1. Introduction

Lateral epicondylitis, also known as “tennis elbow,” is a musculoskeletal disorder of the common forearm extensor tendon, often caused by overuse or repetitive use of the forearm extensor muscles (mainly the short radial carpal extensor) or by direct trauma to the epicondyle.^[1]^The clinical presentation is mainly based on reduced grip and upper limb strength and pain in the lateral aspect of the elbow joint. Current research data shows that the incidence of humeral lateral epicondylitis is around 40%, with an annual incidence of 1% to 3% of the global population and the highest incidence in people aged 35 and over, making it a major public health problem that threatens people’s quality of life.^[2,3]^

The current common treatment methods are mainly surgical and nonsurgical conservative therapies, where nonsurgical treatments mainly include shock wave, corticosteroid injection, platelet-rich plasma injection, and Dextrose prolotherapy therapy nonsurgical rehabilitation treatments. However, there is no standard treatment protocol and most clinicians choose their treatment protocols based on personal experience, which lacks scientific basis and evidence support and has not been included in international guidelines. This current situation is not conducive to the treatment of patients and the standard management of medical quality. The conclusions of studies comparing treatment efficacy between shock wave, corticosteroid injection, platelet-rich plasma injection, and prolotherapy therapy are controversial, with most studies focusing on limited comparisons and lacking direct and indirect comparisons between them, making it difficult to have a clearer and more comprehensive understanding between efficacy. The study uses a reticulated meta-analysis to directly or indirectly compare the efficacy of shock wave, corticosteroid injection, platelet-rich plasma injection, Dextrose prolotherapy therapy interventions for humeral lateral epicondylitis, and to rank the efficacy of the treatment measures, with a view to providing more comprehensive and reliable evidence-based medicine for the clinical management of humeral lateral epicondylitis.

## 2. Data and Methods

Articles are reported in accordance with The National Institute for Health and Care Excellence Grid Meta-analysis Reporting Specification.^[4]^

### 2.1. Inclusion and exclusion criteria

#### 2.1.1. Study population.

The diagnosis of humeral epicondylitis is made on X-ray, computed tomography, or magnetic resonance imaging and in combination with clinical symptoms; age 30 to 80 years; duration of disease > 6 months; and visual analog scale (VAS) for pain > 5.

#### 2.1.2. Interventions.

Include at least 2 of the different interventions of shockwave, endostatin injection, platelet-rich plasma injection, and high concentration glucose augmentation therapy. Discontinue all relevant adjuvant medication during the treatment period in addition to the studied interventions.

#### 2.1.3. Outcome indicators.

The effectiveness of different treatments for humeral epicondylitis and there are clear criteria for evaluating effectiveness in the literature.

#### 2.1.4. Exclusion criteria.

Duplicate published literature; Conference papers and letters; Studies with unclear descriptions of Chinese medicine or the addition of other drugs such as hormones and NSAIDs during treatment; Studies with incomplete or incorrect data information and fruitless contact with authors.

### 2.2. Search strategy

Computer searches were conducted for relevant clinical trials in PubMed, VIP, CBM, CNKI, and Wan-Fang databases. The English search terms mainly included “Lateral epicondylitis,” “Platelet-rich plasma,” “shock wave,” “corticosteroid injection,” “prolotherapy therapy,” etc. The search was conducted using a combination of subject terms and free words, linked by the appropriate Boolean logical operators. The search of all databases was limited to the period between January 1, 2016 and January 1, 2022.

### 2.3. Literature screening and data extraction

Two investigators independently screened the literature according to the inclusion and exclusion criteria, extracted data according to a predefined data extraction form, cross-checked the data, and in case of disagreement, agreed through mutual discussion or referred to a third investigator for decision. Data extraction included basic information about the literature (literature number, title, first author, year of publication, etc), study-related information (mean age of patients, gender composition, disease classification, diagnostic criteria, interventions, frequency of interventions, duration of treatment, follow-up time, efficacy evaluation criteria, and data on outcome indicators) and elements related to the risk of bias assessment.

### 2.4. Statistical analysis

Trials with 3 and more arms were first split into all possible combinations of the 2 arms and the evidence network for comparison of each treatment measure was plotted. Produce comparison-corrected funnel plots to evaluate the intervention for small sample effects or publication bias. Inconsistency factors and their 95% confidence interval were calculated to evaluate the consistency of each closure, with the lower 95% confidence interval equal to 0 considered as good consistency, otherwise the closure was considered to have significant inconsistency. Sensitivity analysis was performed using the MCMC fixed effects model to evaluate the stability of the study results, with the same parameter settings as the random effects model. SUCRA graphs were plotted to predict the ranking of the efficacy of each treatment measure, with a larger area under the curve (0–100%) indicating a better treatment measure. The above graph plotting was performed using Stata 14.0 software.

## 3. Results

### 3.1. Literature search results

**Figure 1.**
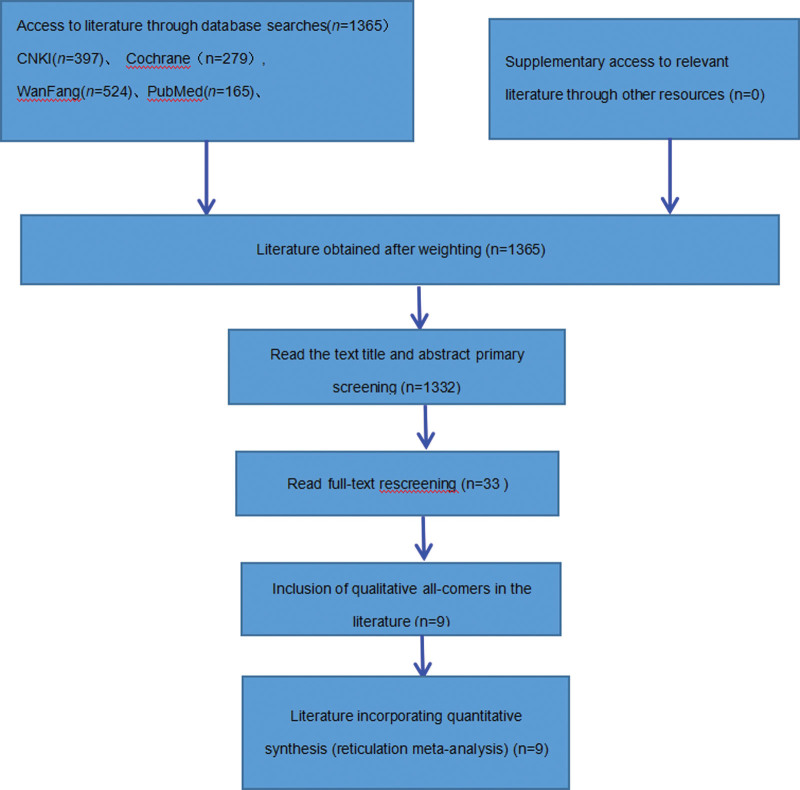
Flow chart of article screening and selection process.

The databases were initially screened for 1365 titles and abstracts, and 9 randomized controlled trials (RCTs) were finally included after the initial screening of titles and abstracts and re-screening by reading the full text.^[5–13]^ The flow chart of literature screening is shown in Figure 1.

### 3.2. Basic characteristics of the included literature

A total of 289 patients with clinically confirmed osteoarthritis of the knee were reported in 9 studies, all with a mean age >42 years, all reporting comparable or nonsignificant differences in age, sex, duration, and severity of disease between groups, with sample sizes ranging from 8 to 32. 1 study was a 3-arm trial,^[13]^ the others were 2-arm trials. A total of 6 combination therapies involving shock wave, corticosteroid injection, platelet-rich plasma injection, Dextrose prolotherapy therapy, and combinations between them were used (see Table 1).

**Table 1:** Basic information of the study.

Inclusion in the study	Treatment group 1			Treatment group 2			Ending indicators
	Interventions	Number of cases (male/female)	Age (yr)	Interventions	Number of cases (male/female)	Age (yr)	
Beyazal and Devrimsel (2015)	C	32(6/26)	**42.6 ± 6.6**	S	32(4/28)	**38.7 ± 9.1**	VAS
Ahadi et al (2019)	P	17(6/11)	46.65	S	16(4/12)	47.25	VAS
Bayat et al (2019)	C	14(3/11)	50.7 ± 7.5	P	14(6/8))	46.2 ± 6.4	VAS
Alessio-Mazzola et al (2018)	PRP	31(18/13)	46.3 + 10.1	S	32(13/19)	50.4 ± 7.3	VAS
Ankit (2016)	PRP	33(-)		C	50(-)		VAS
Carayannopoulos et al (2011)	P	8(-)	49 ± 5.6	C	9(-)	46 ± 5.3	VAS
He et al (2020)	C	15(1/14)	53.2 ± 6.9	P	16(3/13)	58.5 ± 3.4	VAS
Liu et al (2021)	PRP	32(14/18)	51.44 ± 6.59	C	32(17/15)	52.56 ± 5.25	VAS
Zhang et al (2021)	C	16(11/5)	67 ± 14	CS PS	16(10/6) 15(10/5)	65 ± 14 66 ± 13	VAS

C = corticosteroid, CS = corticosteroid + shock wave, P = dextrose prolotherapy, PRP = platelet-rich plasma injection, PS = platelet-rich plasma injection + shock wave, S = shock wave, VAS = visual analog scale.

### 3.3. Evidence network diagram

**Figure 2.**
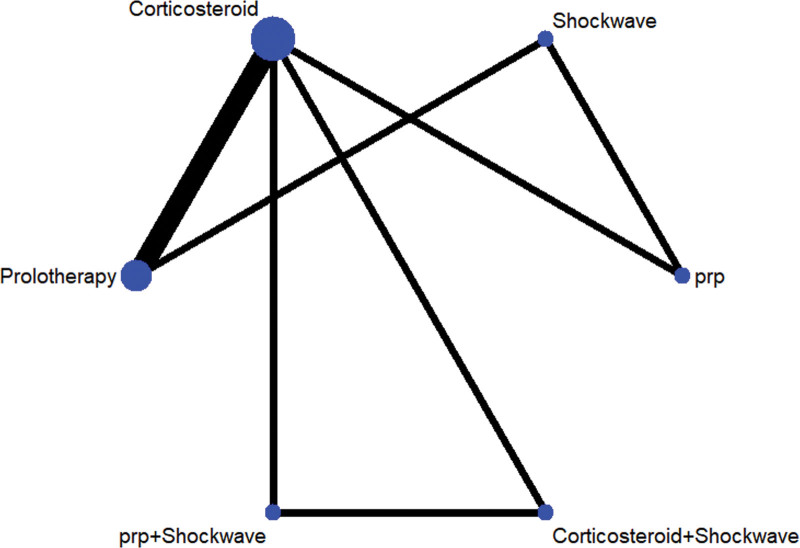
A network meta-analysis of the efficacy of 6 interventional strategies for the treatment of lateral epicondylitis. Note: Lines between points indicate direct comparative evidence of the 2 interventions, no lines indicate no direct comparative evidence and lines indicate no direct comparative evidence. PRP = platelet-rich plasma injection.

Four treatment measures, which could result in 12 different two-by-two comparisons. A total of 6 direct comparisons exist for the 9 included studies, with no direct research evidence for the remaining 6 comparisons whose efficacy comparisons will be generated by indirect comparisons from a reticulated meta-analysis. Figure 2 shows a network diagram of the evidence for the 4 treatment measures for the 9 included RCTs. In the figure, there are connecting lines between points indicating direct comparative evidence for the 2 interventions and no connecting lines indicating no direct comparative evidence.

### 3.4. Contribution of the 6 interventions to the results of the mesh meta-analysis

**Figure 3.**
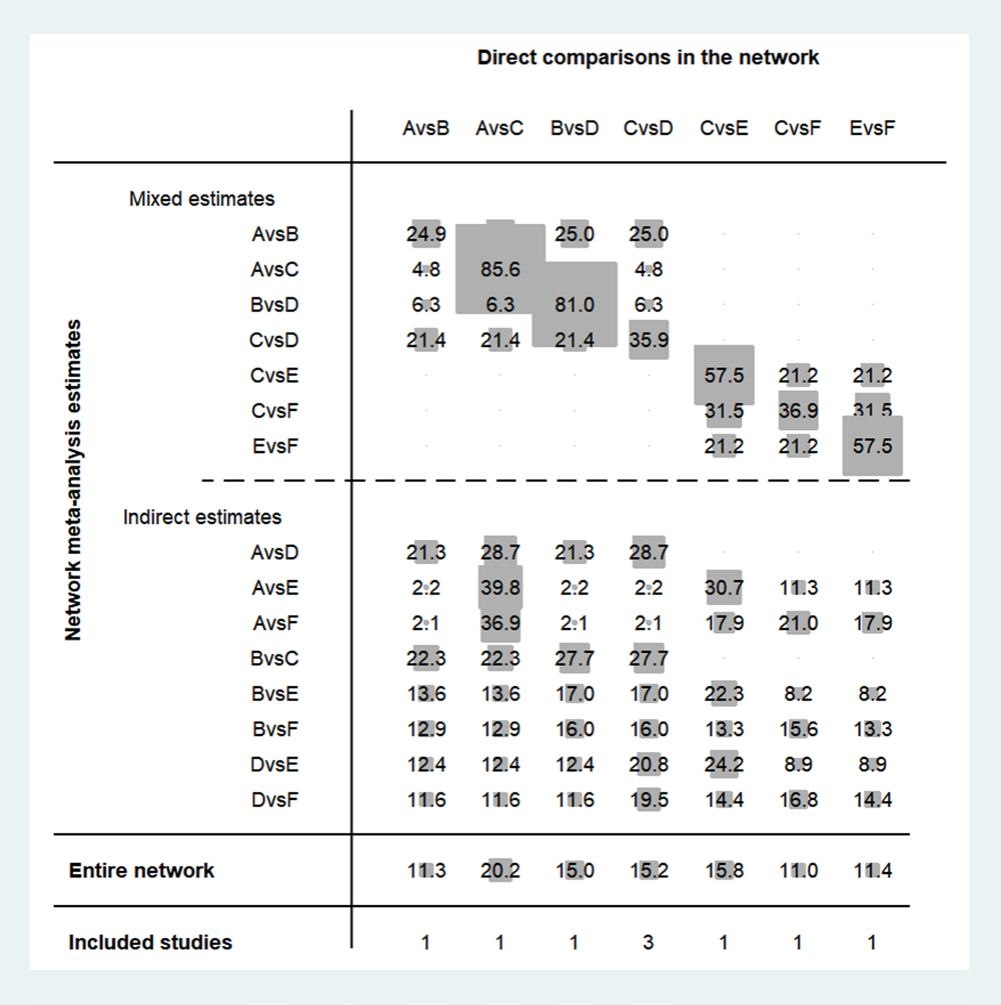
Contribution of the results of the net meta-analysis of the 6 interventions. Note: A: Prp = platelet-rich plasma injection. B: shockwave. C: corticosteroid. D: dextrose prolotherapy. E: platelet-rich plasma injection + shockwave. F: corticosteroid + shockwave.

The impact and contribution of each direct comparison to the network meta-analysis were further analyzed, and the value of the contribution of each group of direct comparisons to the study was expressed as grey circles and weight scores. Figure 3 shows the impact of different direct comparisons on the results of the mesh meta-analysis and the results of the whole network mesh meta-analysis in this study, whose results suggest that for the whole network meta-analysis, the direct comparison of platelet-rich plasma injection with corticosteroid control had the highest contribution (20.20%), followed by the direct comparison of corticosteroid with platelet-rich plasma injection + shockwave for direct comparison (15.8%)

### 3.5. Inconsistency testing

**Figure 4.**
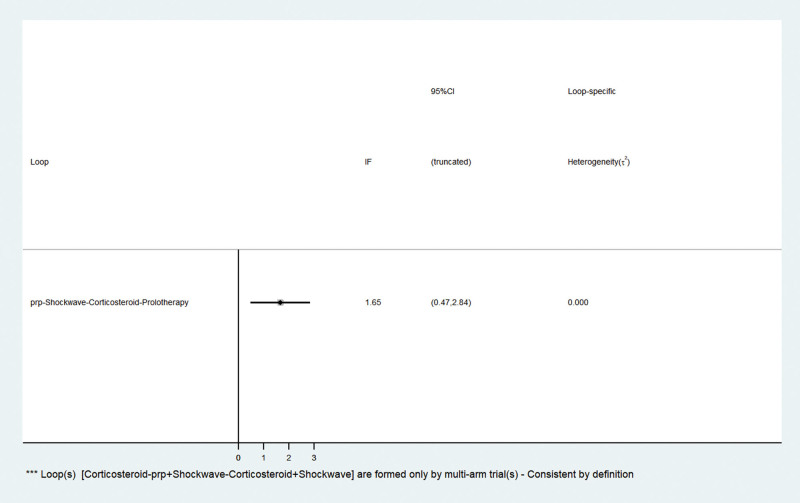
Contribution map of the results of the mesh meta-analysis of interventions. prp = platelet-rich plasma injection.

The results of the inconsistency test showed a global inconsistency test *P* = .0063 < 0.05, indicating good inconsistency. Figure 4 shows 1 quadrilateral ring to test the inconsistency of each closed-loop study finding, and the results show that the inconsistency factor was bounded at 1.65, with a lower 95% confidence interval of 0.47 and 2.84 for both, which in summary indicates that the formation between the treatment measures in this study indicates that each closed-loop inconsistency was good.

### 3.6. Small sample effect detection

**Figure 5.**
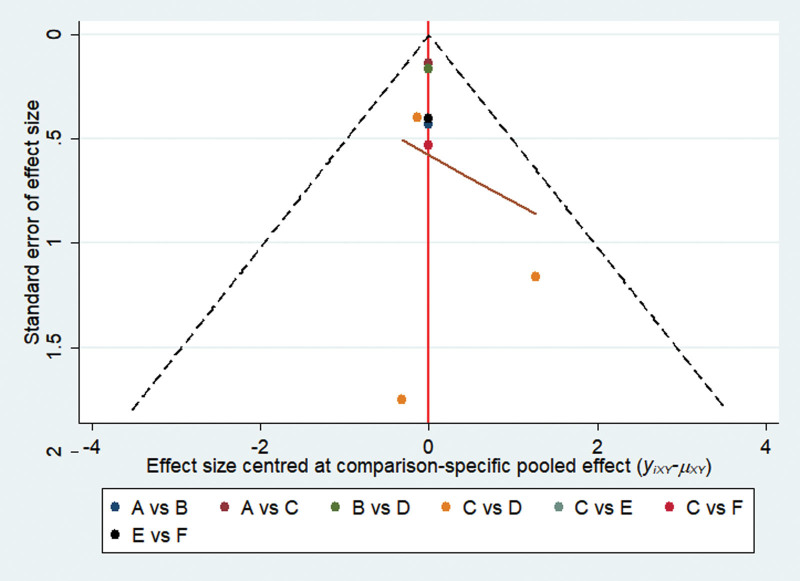
A comparison-adjusted funnel plot. A: Prp = platelet-rich plasma injection. B: shockwave. C: corticosteroid. D: dextrose prolotherapy. E: platelet-rich plasma injection + shockwave. F: Corticosteroid + shockwave.

A comparison-corrected funnel plot of the included studies is shown in Figure 5, where different colored points indicate different direct two-by-two comparisons and the number of points of the same color indicates the number of such two-by-two comparisons in the original study. If the funnel plot is symmetrical, there is no significant small sample effect or publication bias. Figure 4 shows that the funnel plot is generally symmetrical, indicating that there is little likelihood of a small sample effect or publication bias in the study.

### 3.7. Ranking the efficacy of treatment measures

**Figure 6.**
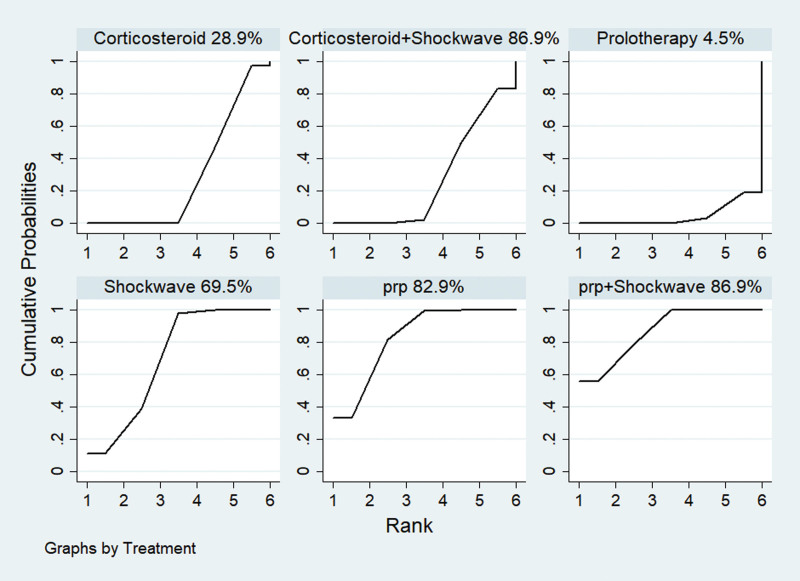
Efficacy of treatment measures SUCRA graph. Prp = platelet-rich plasma injection.

The SUCRA curve was plotted according to the results of the MCMC method random effects model comparison, and the area under the curve was used to predict the ranking of efficacy. Figure 6 shows that platelet-rich plasma injection + Shockwave was the first with an area under the curve of 86.9%. the ranking of the 6 treatment measures was: platelet-rich plasma injection + shockwave > platelet-rich plasma injection > shockwave > corticosteroid > corticosteroid + shockwave > Dextrose prolotherapy.

## 4. Discussion

The treatment of humeral lateral epicondylitis is mainly divided into surgical treatment and nonsurgical rehabilitation treatment. The study found that the success rate of nonsurgical treatment can be as high as 90%,^[14]^ but there are still 4.0% to 25.0% of patients with poor clinical effect on conservative treatment, easy to relapse, produce refractory pain, and some patients even need surgical intervention, which seriously affected the patients’ daily life. A number of studies have found that shock wave, corticosteroid injection, platelet-rich plasma injection, and Dextrose prolotherapy therapy are being used adequately in the nonsurgical management of humeral lateral epicondylitis, but due to the lack of standardization of the various treatments, most clinical use relies on personal experience in the selection of methods. It is difficult to determine which treatment is the most effective, and there is no consensus on the best treatment. The study uses a reticulated meta-analysis to directly or indirectly compare the efficacy of shock wave, corticosteroid injection, platelet-rich plasma injection, Dextrose prolotherapy therapy interventions for humeral lateral epicondylitis, and to rank the efficacy of treatment measures, addressing the lack of direct or indirect comparisons in the original study, which made it difficult to determine differences in the efficacy of different therapies and providing more comprehensive and reliable clinical treatment for humeral lateral epicondylitis. It provides more comprehensive and reliable evidence for the clinical management of humeral lateral epicondylitis. The study included shock wave, corticosteroid injection, platelet-rich plasma injection, Dextrose prolotherapy therapy available in the database for the treatment of 9 RCTs for external humeral lateral epicondylitis, with a combined sample size of 289 cases containing 6 treatment regimens, and extracted the VAS scores from the data, showing the best efficacy in all platelet-rich plasma injection combined with shockwave and the best platelet-rich plasma injection in monotherapy.

The majority of studies have concluded that most lateral epicondylitis of the humerus is due to a chronic aseptic inflammatory reaction at the origin of the tendon of the extensor carpi radialis brevis (100%), the lower surface of the extensor carpi radialis longus, and the anterior border of the extensor digitorum communis (35%). Chronic aseptic inflammatory reaction due to degenerative changes in the tendon tissue caused by repeated stretching and irritation of the digitorum communis (35%), followed by localized muscle tissue congestion and edema and calcification, resulting in painful stiffness and limitation of movement of the elbow joint.^[15]^The pathological basis is a small tear in the tendon caused by repetitive stretching.^[16]^ Platelet-rich plasma is a concentrated product of platelets obtained by centrifugal enrichment in the periphery of the body, which contains a large number of growth factors that promote cell proliferation, differentiation, and repair. It is now widely used in the field of articular cartilage injuries and tendon injuries. It has been shown that platelet-rich plasma improves the clinical symptoms of extrahumeral lateral epicondylitis and promotes the repair of tendon tissue.^[17]^ Clinical treatment with corticosteroid injections is an effective method of conservative clinical treatment with antiinflammatory and analgesic effects. Local injections can provide short-term pain relief and functional improvement, but repeated injections over a long period of time can easily lead to tendon adhesions and degeneration, increasing the risk of infection and further recurrence.^[18]^ Dextrose prolotherapy therapy involves the injection of high concentrations of glucose to induce a local inflammatory response, fibroblast proliferation, increased local growth factor release, and promotion of collagen synthesis, leading to the strengthening of ligaments and tendons.^[19]^Ahadi et al^[20]^ administered hypertonic glucose injections to patients with humeral lateral epicondylitis and found significant improvements in elbow pain, grip strength, and limitation of movement after 4 and 8 weeks. Shockwave is an emerging physiotherapy treatment for bone and muscle disorders. Studies have shown that shockwaves can stimulate the expression of angiogenesis-related growth factors to induce new blood vessel formation and promote tissue cell proliferation for the purpose of tendon or tissue repair.^[21]^ Kan et al^[22]^ evaluated the efficacy of shockwave treatment for humeral lateral epicondylitis using VAS at 1, 3, and 6 months, and the Roles-Maudsley tennis elbow evaluation system, and found that shockwave improved elbow pain and mobility in patients with humeral lateral epicondylitis.

In the meta-analysis of this study, it was found that among these single therapies, platelet-rich plasma injection was more effective than single endostatin injection, augmentation therapy, and shockwave therapy, while platelet-rich plasma injection combined with shockwave was found to be the most effective, which may be related to the fact that platelet-rich plasma injection has abundant growth factors to promote the repair of local and tendinous tissues, but the repair of tendons often takes more than 3 months, while shockwave therapy can effectively provide immediate relief of joint pain and improve joint function.^[23]^ Therefore, platelet-rich plasma injection alone is often less effective than platelet-rich plasma injection alone for short-term pain relief in the treatment of humeral lateral epicondylitis, and platelet-rich plasma injection combined with shockwave therapy is more effective than platelet-rich plasma injection alone for the treatment of humeral lateral epicondylitis, and therefore it is considered that platelet-rich plasma injection combined with shockwave therapy may be preferred in the clinical practice of humeral lateral epicondylitis.

The lack of a direct comparison with the incorporated platelet-rich plasma injection combined with shockwave therapy vs other therapies affects the comprehensiveness of the evaluation of the efficacy of humeral lateral epicondylitis. In practical clinical use, these treatments may be more effective than those included in this study, and there is a need for more comprehensive clinical studies to provide more reliable evidence for treatment options for humeral lateral epicondylitis. However, no significant asymmetry was found in the study comparison-corrected funnel plots, suggesting no significant small sample effects or publication bias, and inconsistency tests suggested good agreement across the closed loops, indicating stable results for these 6 measures of humeral lateral epicondylitis treatment.

In summary, reticulated meta-analysis provides a more systematic and objective evaluation of the effectiveness of shock wave, corticosteroid injection, platelet-rich plasma injection, and Dextrose prolotherapy therapy in the treatment of humeral lateral epicondylitis and facilitates the selection of the best treatment option for humeral lateral epicondylitis. The results concluded that platelet-rich plasma injection combined with shockwave therapy was the best of the 6 nonsurgical treatments included, and that in actual clinical practice, particularly for refractory humeral lateral epicondylitis, platelet-rich plasma injection combined with shockwave therapy could be preferred based on the findings of this study and the physicians’ own experience. Based on the shortcomings of the existing studies, the conclusions of this study still need to be confirmed by a large number of well-designed and appropriate RCTs that cover a wide range of Chinese medical treatments.

## Author contributions

**Conceptualization:** Qing wan, Xingzhen Lin.

**Data curation:** Fang Zhi, Xingzhen Lin

**Formal analysis:** Qin Lan, Xingzhen Lin.

**Investigation:** Xingzhen Lin

**Resources:** Xingzhen Lin, Fang Zhi

**Software:** Xingzhen Lin.

**Supervision:** Fang Zhi, Qin Lan.

**Writing – original draft:** Xingzhen Lin, Qin Lan.

**Writing – review & editing:** Xingzhen Lin, Qing wan.

**Conceptualization:** Xingzhen Lin.

**Data curation:** Xingzhen Lin.

**Formal analysis:** Qin Lan, Xingzhen Lin.

**Funding acquisition:** Xingzhen Lin.

**Investigation:** Xingzhen Lin.

**Resources:** Fang Zhi.

**Software:** Xingzhen Lin.

**Supervision:** Qin Lan.

**Writing – original draft:** Qin Lan, Xingzhen Lin.

**Writing – review & editing:** Qing Wan.
